# Early-life adversity in rodents: Experimental design is of the essence

**DOI:** 10.1016/j.ynstr.2026.100813

**Published:** 2026-04-16

**Authors:** Rixt van der Veen, Marian Joëls, Aniko Korosi

**Affiliations:** a Swammerdam Institute for Life Science, Center for Neuroscience, Brain Plasticity Group, University of Amsterdam, the Netherlands; b Center for Urban Mental Health, University of Amsterdam, the Netherlands; c University Medical Center Groningen, University of Groningen, the Netherlands

**Keywords:** Early-life stress, Rodent, Timing, Sex, Brain

## Abstract

Early-life adversity (ELA) is a well-documented risk factor for the development of psychiatric illnesses. Prospective investigations in humans are complex and limited in their ability to study the underlying neurobiological mechanisms, which is why many have reverted to animal models to examine the lasting consequences of ELA. In this contribution, we focus primarily on exposure to ELA during the postnatal period and highlight crucial elements of the experimental design that decisively contribute to the observed outcome. These elements include i) aspects of timing, encompassing the developmental stage during which ELA is experienced, the duration and repetitiveness of early-life stressors, and the time point at which the outcomes are investigated; ii) the early-life environment, e.g., the quality and quantity of parental care, the breeding specifics, housing conditions and nutrition, or mitigating interventions; iii) characteristics of later-life readouts, i.e., specific behavioral domains examined, the tasks selected to probe these domains, and the state of the animal at the time of testing; and iv) sex differences. We conclude by discussing how to maximize the advantages of ELA animal models to gain a comprehensive insight into the (neuro)biological mechanisms underlying the lasting consequences of ELA.

"Design is intelligence made visible." -- Alina Wheeler

## 1 Introduction

A striking proportion of children face early-life adversity (ELA), with recent estimations of 30-35% experiencing multiple adversities under the age of 18 (Madigan et al., 2025). This involves a wide range of conditions, such as family dysfunction, parental separation, and emotional or physical neglect and abuse. ELA is a well-documented risk factor for individuals’ lifetime risk of mental disorders and is associated with earlier disease onset, more severe symptomatology, and treatment resistance (Lippard and Nemeroff, 2023). A recent umbrella review revealed that, globally, the chances of developing later-life mental health problems are 66% higher among individuals who experienced ELA (Abate et al., 2025). For conditions like depression and anxiety, this translates into a lifetime risk of 28% compared to 17% in the general population. Despite these associations, it is important to realize that the vast majority of individuals do not develop psychopathology after ELA, pointing to innate or exogenous factors that can mitigate the risk (see also section 3). Indeed, childhood is a highly adaptable period during which interventions can have a major beneficial impact, too (Han et al., 2025). Gaining insight into the (neurobiological) mechanisms contributing to the (long-lasting) impact of ELA is thus not only essential for identifying targets for intervention, but also to pinpoint windows of opportunity for such interventions (Bakermans-Kranenburg and van IJzendoorn, 2015; Cattaneo et al., 2024; Heim et al., 2019).

Establishing causal links between ELA and psychopathology later in life and delineating the underlying pathways are daunting tasks in humans. For one thing, cross-sectional approaches suffer from a recollection bias, while prospective and longitudinal investigations are lengthy and expensive, given the long time lag between ELA and the manifestation of clinical symptoms, which also poses a compliance issue. Moreover, investigations in humans are complex due to numerous gene-by-environment interactions, over which there is no (or only restricted) control. Furthermore, the possibilities for investigating the underlying neurobiological mechanisms in detail are limited. Therefore, many have reverted to rodent models to examine whether and how ELA affects mental health in the longer term (Knop et al., 2017; Waters and Gould, 2022).

The topic of ELA and its consequences in animal models has been amply reviewed (e.g. Bath, 2020; Birnie and Baram, 2025; Cattaneo et al., 2024; Luby et al., 2020; Murthy and Gould, 2020; Reemst et al., 2022b; Speranza et al., 2024). Here, we introduce a new angle by discussing several key components of the experimental design that will critically influence the outcome. In our contribution, we will focus on ELA exposure during the early *post*natal phase. Considering that the dam's presence and behavior are crucial elements in the pups' environment during the first postnatal week, most ELA rodent models are based on a disruption of the mother-pup interaction. The most commonly used rodent models targeting the first postnatal week(s) are (see Box 1): i) maternal separation (MS) (Tractenberg et al., 2016), ii) maternal deprivation (MD) (Ferreira de Sá et al., 2023), iii) models of scarcity, e.g. by housing the dam and her litter with limited bedding and nesting material (LBN) (Walker et al., 2017), iv) unpredictable stress of the mother (Weiss et al., 2011), or v) combinations thereof, e.g., maternal separation combined with LBN (Peña et al., 2019b). We focus on these dominant paradigms because they have supplied ample data, allowing for a critical perspective and distilling generalizable concepts of the design. As a result, our contribution is not providing a comprehensive overview across all existing models. In the following sections, we will argue that specific aspects of the experimental design in rodent ELA studies are critically important for the outcome. This is much in the spirit of the late Seymour (Gig) Levine, one of the founding fathers of the field of early-life research (Levine, 1957), who often said that a well-thought-out design makes up 90% of a successful experiment. The aspects that we will discuss focus on i) the importance of timing; ii) environmental factors during early-life and post-ELA; iii) characteristics of later-life readouts; and iv) sex differences. Rather than providing a comprehensive review of the literature, we will highlight these four aspects with a selection of examples that illustrate how insight into the consequences of ELA may depend on critical experimental choices.

Box 1 **Common rodent models for postnatal adversity and their translatability**

**Figure:**
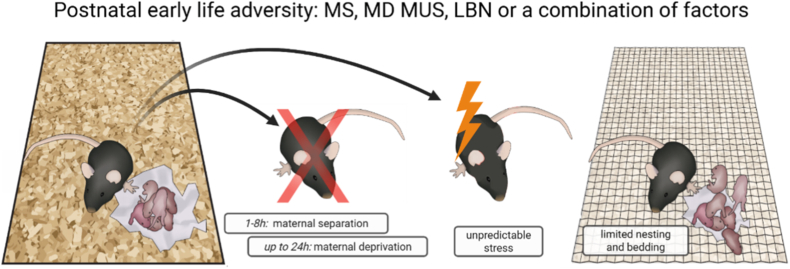

Figure Box 1: This review focuses on postnatal models of early-life adversity involving changes in maternal care. These models include separation of the pups from their mother by taking either the mother or the pups from the homecage for 1-8h daily (maternal separation, MS), or up to 24h (generally called maternal deprivation (MD), exposing the mothers to unpredictable stress (MUS), providing limited nesting and bedding material (LBN), or a combination of these factors.

The postnatal rodent models discussed in this paper are hypothesized to represent deprivation, neglect, and unpredictability of parental care toward the pups. Effects on cognitive function, anxiety, reward-related tasks, and social behavior have been found with several models, pointing to a general developmental effect of these models instead of targeted effects on specific outcomes. There seem to be a species effects, with rat offspring showing larger effect sizes compared to mice offspring. A combination of models and/or second hits like purchased pregnant dams (cumulative risk) induces stronger effects.

This is comparable to the human literature, where the specificity of conditions has been claimed, with distinct developmental mechanisms influenced by different forms of adversity, but this is very difficult to establish firmly, since most adversities are not unidimensional and cause collateral effects. The cumulative risk has also been demonstrated in human studies, counting the number of discrete adversity exposures, having an additive effect on the outcome. More recently, a dimensional approach has been postulated, where, next to specific or cumulative risk, different dimensions of adversity might affect different behavioral domains later in life. Certain types of ELA exposure might predominantly represent deprivation, others threat and harshness, and yet others unpredictability, or a combination of these (McLaughlin et al., 2021). In this dimensional approach, deprivation experienced early in life would be mostly linked to changes in cognitive development. Conversely, threat and harshness experiences are thought to be mostly associated with alterations in emotion processing, whereas unpredictability would favor a need for short-term rewards and intense monitoring of environmental changes (e.g., attention shifting). The ELA rodent models cover all these dimensions, but, for now, have not clearly pointed to distinct effects in specific outcome domains. As discussed in the main text, with our short-lasting, sporadic experimental tests, we could easily miss long-term behavioral measures, but it might also be that, with our rodent studies, we are limited in modeling the finer nuances of human (mental) health and behavior.

Rodent models have the advantage of excellent control over the genetic background and environment, short generations, and the possibility of using invasive methods when studying neurobiological processes (Knop et al., 2017). Clearly, they also have many limitations, including the differences in lifespan, the pace of brain development, and, next to similarities, also differences in brain morphology and networks (Beauchamp et al., 2022; Luby et al., 2020). Moreover, there are important differences in social context and environmental experiences to which organisms are exposed (the ‘exposome’), and limitations to study aspects of psychopathology (Barroca et al., 2022).

It was not without reason that Gig Levine worked with both rodent and primate models to understand the effects of early-life experiences on stress resilience and vulnerability (Suchecki, 2018). Specifically the role of maternal care in shaping stress responses and uncovering the mechanisms of attachment behavior can be studied in much more detail in primates than in rodents (Cirulli et al., 2009; Savidge and Bales, 2020; Suomi, 2005). Primates share the more complex social relationships of humans and their physiological consequences (Levine et al., 1997), as well as a more comparable pace in brain development, brain morphology, and neuronal networks (Yokoyama et al., 2021). We should thus be careful not to project findings in rodents too readily to (typically) human processes, but preferably use them as basic proof-of-principle studies pointing to mechanisms worthy of exploration in primates and ultimately in humans.

Alt-text: Box 1

## 2 The importance of timing

Careful timing is important in many ways when conducting research, in this paragraph we highlight three aspects of timing that one should pay attention to when designing early life adversity models. These are related to timing of the model during the developmental stage of the pup and the crucial role of maternal care (section 2.1.), the timing characteristics of the early life adversity, like duration, repetitiveness and time of day (section 2.2), and the moment at which the sequelae of ELA are investigated (section 2.3). We are aware that timing is a broad concept and not all aspects of it are covered within this selection.

### 2.1 Timing of ELA exposure during developmental stage and the crucial role of maternal care

The consequences of early (postnatal) life manipulations depend on the developmental stage at which they are experienced. Thus, depending on the functional networks and domains of interest, the specific time windows during which the developmental trajectories of these functional networks are potentially affected by ELA need to be taken into account. These developmental stages go hand in hand with mother-pup interaction, explaining the importance of maternal care and the effects of interfering with it. During the first postnatal period of a pup's life, its behavior is geared toward approach and attachment, involving minimal amygdala involvement and minimal avoidance when encountering new environmental cues (Sullivan and Opendak, 2021). Around the time rodent pups develop fur and start to crawl (∼P10), the amygdala begins to functionally develop and support fear reactions to environmental cues, promoting a balance between approach and avoidance behaviors. Slowly, the pup transitions from mother-oriented to more peer-oriented behavior, while in the meantime, the amygdala-prefrontal cortex connectivity develops and feedback regulation of the HPA axis is put in place, enabling the adolescent rodent to deal with external stressors more independently. Until this happens, the dam buffers environmental influences, maintaining the pups' so-called stress (or rather, adrenal) hyporesponsive period (P1-12 in mice, P3-14 in rats) (Cirulli et al., 1994; Levine, 1994). Interfering with the mother's care and availability before the pups' own stress and fear responses are in place threatens the buffering provided by the mother. Such interference is a key element of many commonly used (postnatal) ELA models. Indeed, studies using maternal separation models have clearly demonstrated compromised stress buffering, showing that the mother's absence increases stress hormone levels and sensitizes mice to subsequent low-intensity stressors (Enthoven et al., 2008; Schmidt et al., 2002). A study conducted in rats nicely demonstrated the influence of timing here, since 24h MD at P3 *increased* CORT-induced ACTH release at P20, whereas 24h MD at the end of the hyporesponsive period at P12 *attenuated* the ACTH response at P20, with in-between results in the case of 24h MD at P7 (van Oers et al., 1997).

Another example of the importance of ELA timing during development is a study in which the effect of LBN on social behavior was investigated, this showed that LBN conditions early in postnatal life (P2-P12) did not affect social defeat outcomes in mice, while the same procedure later on (P10-P17), when social behavior is developing, rendered adult mice more susceptible to depression-like behavior after social defeat (Peña et al., 2017). Thus, timing is of relevance with respect to the distinct developmental trajectories of specific rodent brain regions and circuits, keeping in mind that rat brain development is slightly slower (∼1-2 days) compared to mice (Workman et al., 2013). From a translational point of view it is important to note that although the sequence of key events in brain maturation is largely consistent between humans and rodents, it is of course, crucial to keep the cross-species differences in time scale of developmental trajectories in mind (Luby et al., 2020; Semple et al., 2013).

### 2.2 Duration, repetitiveness, and time of day of ELA

Timing is also a relevant aspect of the intervention models themselves. For instance, the duration of (daily) ELA exposure is important for later-life outcomes. For instance, 1h MS -which seems closer to a handling protocol than to ELA-was found to generally *enhance* synaptic plasticity in the adult hippocampus, whereas a 6h MS paradigm appeared ineffective (Derks et al., 2017). More chronic conditions, such as those caused by LBN, *reduced* hippocampal synaptic plasticity (Derks et al., 2017). In a large meta-analysis of the behavioral consequences of ELA, a 10-day ELA protocol was found to cause maximal effect sizes (Bonapersona et al., 2019). Whether this is true for other outcome parameters needs further investigation.

Models like MS use repetitive stress exposure during the postnatal period. Exposing rodents to homologous stressors on sequential days contains an element of predictability and learning. For instance, when CD1 mice were exposed to 8h MS on three consecutive days (P3-5), they showed a separation-induced corticosterone response on the first day, but the response to separation decreased over subsequent days (Enthoven et al., 2008). Yet, the corticosterone response to a novel mild stressor was sensitized in these animals, revealing the impact of this 8h MS model. The habituation to homotypic stressors may be species-dependent: Mice seem to adapt better to maternal separation than rats. In fact, while maternal separation in rats is associated with increases in anxiety-like behavior, no clear effects could be observed in mice studies (Wang et al., 2020). Indeed, MS models up to 3h may not be very effective in mice (Millstein and Holmes, 2007; Savignac et al., 2011). It has been hypothesized that the increase in maternal care upon reunion serves as a protective factor against the effects of repeated separation. When unpredictability was increased by applying the 3h MS protocol at random time points in combination with unpredictable stress exposure of the mother, maternal behavior in mice was disturbed upon reunion, and behavioral consequences in the offspring were stronger (Jaric et al., 2019; Weiss et al., 2011). This species effect does not seem to be unique to the MS model as in general ELS effects in rat offspring show larger effect sizes compared to mice offspring (Bonapersona et al., 2019).

Different recourse scarcity models have also shown that changes in implementation and frequency can account for a differential impact on maternal care, that is, continuous LBN on a mesh wire gives rise to fragmented and unpredictable care, with little change in overall duration of care of specific aspects of care, while a version without the wire mesh needing frequent cleaning or an intermittent version with dams not habituated to the impoverished environment gives rise to abusive and potentially harmful behaviors such as stepping on, dropping and rough handling of the pups, leading to differential outcomes (Walker et al., 2017).

Notably, timing in the most literal sense - i.e., the time of day when pups are exposed to ELA-also influences the later-life outcome (de Souza et al., 2022, in rats; Parfitt et al., 2007, in mice).

### 2.3 Timing in relation to the moment of examining the consequences of ELA and the outcome of interest

Finally, timing is also relevant when selecting the moment at which the sequelae of ELA are examined. The nature of ELA-induced effects may take different forms during different life stages. For instance, ELA between P8 and P12 revealed infant rat social behavior deficits, which were followed by adolescent depressive-like behaviors (Raineki et al., 2012). This could be explained by developmental changes in the brain, as well as the time lag between the moment of ELA and the observable effects.

It is important to realize that some of the ELA sequelae may be apparent only during particular windows in time, which also might (but not necessarily always does) provide insight into sensitive windows for interventions. For instance, mice raised in LBN conditions show a reversal of GABA-induced depolarization into hyperpolarization between P6 and P9, whereas this occurs only around P15 in controls (Karst et al., 2023). This finding is in line with the frequently observed acceleration of rodent brain maturation after ELA (Bath, 2020). Eventually, though, the control and ELA groups are comparable, as the ELA and control groups were indistinguishable at P21. By limiting investigations to P21, one might have inadvertently concluded that ELA has no effect. The reverse can also occur: Certain ELA consequences may only emerge after a delay, so that investigations before this point in time would lead to the (wrong) conclusion that ELA does not affect the organisms. For instance, at the level of the neuroimmune system, ELA does not lead to differential gene expression in mouse hippocampal microglia at P9, but does cause substantial changes in the gene expression profile at P200 (Reemst et al., 2022b).

### 2.4 Concluding statement about the importance of timing

Choices regarding i) the developmental stage (brain, behavioral, and neuroendocrine) during which exposures take place, ii) the timing aspects of the exposure model itself, and iii) the time lag, and the moment in life during which the consequences of ELA are examined all contribute to the observed outcome. Varying these aspects—e.g., by exposing rodents at different ages or for different durations to ELA or taking multiple time points into account when investigating the later-life consequences of ELA, all within the same experimental series—will yield more complete insights.

## 3 The importance of environmental factors and possibilities for interventions

**Fig. 1:**
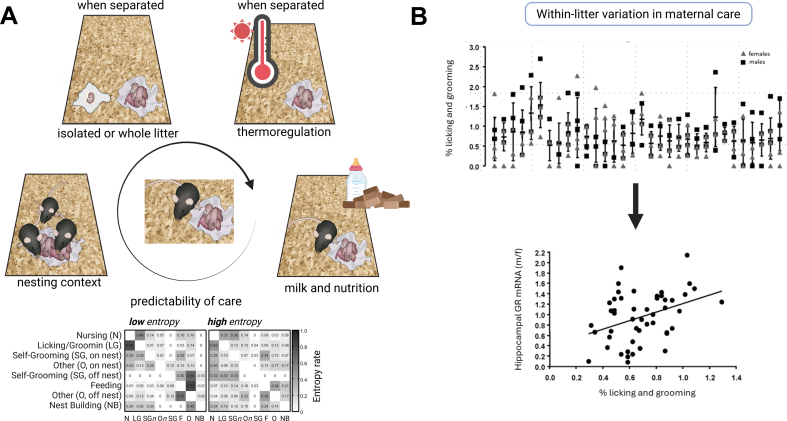
A. Cartoon of peri- and post-ELA conditions that contribute to later-life outcomes, next to the adversity exposure. Relevant factors include nutrition, breeding conditions of earlier generations (e.g communal nesting) and quality and quantity of maternal care (including predictability). The paradigm of separation (e.g. single or whole litter) and thermoregulation are crucial aspects of the separation design. In natural circumstances, maternal care, nutrition, and thermoregulation are interconnected, but in laboratory settings, they can be varied more independently. At the bottom, a typical example of low (left) and high (right) entropy in a dam's behavior toward her litter in an LBN experiment, corresponding to, respectively, a high and low degree of predictability of maternal care. B. (Top) Even *within* rat litters (each column shows the results of a single litter), the percentage of time dams spent on licking and grooming (%LG) of their pups varies considerably. Observations in this graph were obtained from five 1h observation slots per day (07:00,10:00, 13:00, 17:00, and 20:00 h) during the first week after birth. The behavior of the dam was scored every 3 min, generating 20 observations per observation period and a total of 700 observations per dam for the whole week. Error bars depict one SD above and below the litter mean (horizontal stripe). Male pups are represented by a black square, female pups by a grey triangle. Overall, male pups received significantly more care than females. (Bottom) The amount of individual licking and grooming (%LG) received during the first postnatal week -scored as described above-significantly correlated with total hippocampal glucocorticoid receptor (GR) mRNA expression in young to young-adult male and female rats. Reproduced with permission from Van Hasselt et al. (2012).

As outlined above, the dam's presence and behavior are crucial elements in the pups' environment during the first postnatal week. Interfering with the maternal presence or providing otherwise stressful circumstances deprives the pups of care, in terms of feeding, sensory stimulation (through licking and grooming), evacuation of waste (anogenital licking), and temperature regulation. In this section, we highlight the relevance of maternal care and nesting conditions, nutrition (particularly regarding the composition of the dam's breastmilk), and temperature regulation (section 3.1) (Fig. 1A). Clearly, these are by no means the only environmental factors that might play a role in the eventual outcome. In subsection 3.2, we will discuss peri-ELA or post-weaning interventions related to corticosteroid modulation and nutrition, as well as discuss sensitive time windows for intervention.

### 3.1 Maternal care, nutrition, thermoregulation, and post-weaning environment

*Maternal care*: Maternal care in rats shows a large degree of natural variation in quantity (Champagne et al., 2003), which, in turn, significantly impacts the offspring's brain development. For instance, the brains of litters from high-licking, grooming dams adopting an arched-backed nursing style differ greatly from those of litters from mothers at the opposite end of the spectrum, a process involving epigenetic programming (Peña, 2025; Szyf et al., 2005). Of note, maternal care also displays considerable *within-litter* quantitative variation, with lasting consequences for brain function (see e.g. (van Hasselt et al., 2012); Fig. 1B ). As described above, different ELA models have in common the interference with maternal care, like the lack of care when the mother is temporarily absent. To avoid compensatory care when mothers are reunited with the pups in these models, the amount of stress needs to be above a certain threshold, this might be accomplished by increasing the time of separation to at least 3 h/day (up to 24h separation). The threshold for ELA effects might be higher in mice compared to rats, since ELS effects in rat offspring show larger effect sizes compared to mice offspring (Bonapersona et al., 2019). Indeed, mouse studies on maternal separation often include more intense settings, like exposing the mothers to an additional stress during separation or combining ELA models (George et al., 2010; Peña et al., 2019a; Weiss et al., 2011), moving the litter from the homecage during separation (Peña et al., 2019a) and/or individually housing the pups during separation (Kestering-Ferreira et al., 2021; Zimmerberg and Sageser, 2011).

With respect to the quality of maternal care, predictability is assumed to be a crucial factor. For instance, while in the LBN model the total amount of time spent by the dam on her offspring is, on average, comparable to that of mothers living in standard housing conditions, the degree of unpredictability (i.e., behavioral entropy) of the former is much higher, and found to be predictive of emotional development of the offspring (Molet et al., 2016). It has even been argued that the unpredictability of the dams' behavior is the crucial factor in the translatability of this model to the human situation (Birnie and Baram, 2025; Davis et al., 2022; see also McLaughlin et al., 2021; for more details, see Box 1). The relevance of the dam's (un)predictable nest presence (from the pups' view) may be particularly apparent in laboratory conditions, as mothers are routinely individually housed before parturition, whereas in natural populations, female mice and rats often nest and nurse communally, which may provide protection against conspecific infanticide and ensure more nest presence than in ELA studies (Hayes, 2000; Jo Manning et al., 1995). Importantly, the individual housing in most experimental laboratories also contrasts with the conditions in commercial breeding facilities, where specifically mice (more than rats) are often communally bred (Pritchett-Corning, 2011). This is something to be aware of when instead of in house breeding, researchers order females from commercial breeding facilities for ELA experiments, since communal nesting can enhance sociability and influence maternal care of the offspring (Branchi et al., 2006; D'Andrea et al., 2007; Knop et al., 2020). One should also be aware that when ordering time-pregnant females, the transportation stress can potentiate ELA effects in both mice and rats (Bonapersona et al., 2019)

*Nutrition*: Early-life nutrition is a crucial factor in brain development, as the brain is the fastest-growing organ with high metabolic activity and energy demand (Black, 2018; Cusick et al., 2022; Cusick and Georgieff, 2016). Minor nutritional deficiencies can have far-reaching effects on brain structure and functioning (Georgieff, 2023). The breast milk that a dam provides to her offspring is an important element of the mother-pup interactions. In rodents and humans alike, breastmilk provides the building blocks, microbiome, and bioactive components for growth and (neuro)development. It was shown that stress in lactating mothers directly impacts breastmilk composition of fatty acids (Juncker et al., 2022), amino acids (Juncker et al., 2023), and microbiome (Juncker et al., 2025). In rats, an increase in lactation quality and richer microbiome diversity was observed after enriched housing of breeding dams, posited to be less stressful (DeRosa et al., 2022). In line with this, we have proposed that nutrition in general (Yam et al., 2015) and breastmilk in particular (Reemst et al., 2022c) might play a key role in ELA-induced deficits. This is corroborated by preclinical in vivo mouse studies of ELA, showing that ELA leads to altered nutrient composition of the brain (Geertsema et al., 2025).

*Thermoregulation*: The core temperature of the pups is another relevant aspect of the early-life environment, specifically in the first 10 days, when little fur is present, and metabolism and neuromuscular function are developing. Only at weaning (around postnatal day 21) has the offspring's thermoregulation fully developed (Pritchett-Corning et al., 2015). Consequently, when pups are removed from the dam or the nest during the first postnatal week, placement of the pups on a heating pad (around 30-32 °C) upon separation from the mother is necessary; at older ages, the necessity depends on the length and mode of separation (i.e., as single pups or as a nest, see also Fig. 1A).

*Post-weaning housing conditions*: So far, we have only briefly touched on some environmental aspects up to weaning. However, housing conditions after weaning may also contribute to the later-life consequences of ELA. A recent study nicely demonstrated that rearing environments across different facilities (differing in standard husbandry in terms of type of cages, light/dark regime, presence of background noise, type of diet and enrichment material) influenced mouse phenotypes, both at the behavioral and molecular levels, which could account for different outcomes of the same model across different labs (Jaric et al., 2022). There is indeed evidence that post-weaning housing conditions e.g., through environmental and/or social enrichment (de Sousa Fernandes et al., 2024) are able to counteract the consequences of ELA. On the other hand, we and others have shown that housing rats and mice in complex, enriched cages can lead to a strong “been-there-done-that” individualistic phenotype that, if anything, overshadows ELS effects rather than normalizing behavior (Kentrop et al., 2018; van der Veen et al., 2020). Since enrichment is a term that is used for many changes in the cage—ranging from the addition of an extra gnawing stick to big stimulating cages with multiple peers, additional space, multiple stories, and objects, and everything in between (Cait et al., 2024)—one should be specific about it and cautious about how to use and interpret the effects of enriched environments. In general, it is good to realize that our standard laboratory cages offer little challenge (Mieske et al., 2022), and increasing space and enrichment can increase welfare and meaningful results, but at the same time might bring about a more challenging and sometimes stressful environment (Bailoo et al., 2018; Curley and Champagne, 2023; Kempermann, 2019). In addition, species and strain differences should be taken into account regarding group housing. Indeed, social housing in larger groups can enhance stress coping in female rats, while it can increase adverse effects of stress in male mice (Beery et al., 2020; Tzeng et al., 2017; Westenbroek et al., 2003).

### 3.2 Possibilities for intervention

Among the factors in the early-life environment that can influence developmental programming, glucocorticoid exposure and (mal)nutrition have been well-studied. Of note, both mechanisms are interdependent: e.g. maternal low-protein diets can increase glucocorticoid levels, while excess glucocorticoids can lead to decreased food intake (de Freitas Mathias et al., 2024).

*Impact of ELA on HPA axis and its potential as target for intervention*: Many ELA models result in i) adrenal hypertrophy, ii) increased HPA-responsivity in combination with impaired negative feedback regulation, pointing to glucocorticoid resistance at the level of the pituitary gland, and iii) reduced corticosteroid receptor expression (Babicola et al., 2021; Gore and Gould, 2024; Jorgensen et al., 2025). Further evidence that HPA-axis dysregulation is related to long-term ELA effects stems from studies using mice bred on high,- intermediate-, or low- HPA-axis reactivity. Here, mice with low HPA-axis reactivity were relatively protected from cognitive and behavioral changes following ELA exposure, while high-responders were more impaired (McIlwrick et al., 2016, 2017). However, not all studies using ELA models have replicated changes in HPA-axis activity, and a subset reports a blunted stress response (Jorgensen et al., 2025). Although thoroughly reviewed, a meta-analytical review on the impact of postnatal ELA on HPA axis, such as recently published on prenatal stress in rodents (Creutzberg et al., 2021), is—to the best of our knowledge—still lacking.

An interesting question is whether the environmental factors during the first postnatal week have an impact on peripheral and/or central aspects of the pups’ stress system. This was nicely addressed in a study where rats were exposed to 24h MD at P11 and where the elevation of cort was prevented by the administration of dexamethasone. While cort was indeed reduced at PD12, this did not prevent most of the ELA-evoked central changes (van Oers et al., 1998). The same study found that mimicking tactile stimulation of pups by stroking with a soft paintbrush during MD on PD11, was capable of reversing most of the peripheral and central HPA-axis effects on P12, while only the combination of stroking and supplementary feeding caused a phenotype similar to that of nondeprived rats (van Oers et al., 1998). Moreover it was found that mimicking tactile stimulation prevented decreases in growth-regulating enzymes caused by maternal separation in rats (Kuhn and Schanberg, 1998). Touch interventions have since these early studies also been widely implemented and shown effective in human newborns (Packheiser et al., 2024).

There is more evidence that targeting the HPA-axis can be effective to counteract the impact of ELA. For instance, 3-day administration of the glucocorticoid receptor antagonist mifepristone during early puberty effectively prevented (or normalized) the changes caused by ELA exposure on adult contextual learning in relatively low (Loi et al., 2017) and high arousing (Arp et al., 2016) conditions, although this was not always found (Sanguino-Gómez and Krugers, 2024). Similar beneficial effects were observed following adult mifepristone treatment on the ELS-induced effects in APP-mutant mice on cognitive flexibility (Lesuis et al., 2018). Also, other hormonal systems, like oxytocin, that link with the HPA-axis have received attention as possible targets for intervention (Ellis et al., 2021; Gatta et al., 2018; Jorgensen et al., 2025; Londono Tobon et al., 2018).

*Impact of ELA on brain nutrient composition and its potential as target for intervention*: ELA has been shown to impact on the brain's nutrient composition. For example levels of offspring's brain methionine (Naninck et al., 2017), fatty acid (Yam et al., 2019) and lipidome composition (Reemst et al., 2024) have been shown to be altered after ELA exposure. The potential protective effects of early nutritional interventions against the lasting effects of ELA on cognition have been explored. For instance, early supplementation with fatty acids, methyl-donor micronutrients, and polyphenols can protect against ELA-induced cognitive decline (Geertsema et al., 2025). In particular an early diet increasing the availability of omega 3 long-chain polyunsaturated fatty acids (i.e. low in the ratio of linoleic acid (LA, ꙍ-6)/α-linolenic acid (ALA, ꙍ-3)) provided from postnatal day 2 till postnatal day 42 has been shown to prevent ELA-induced adulthood cognitive impairments in mice and the associated reduction of hippocampal cell survival and increase in phagocytic microglia (Yam et al., 2019). In addition, ELA, as well as the early dietary fatty acid ratio, was shown to alter the brain molecular profile (Reemst et al., 2024), the lipidome, and the oxylipins (polyunsaturated fatty acid derivatives) profiles, potentially contributing to the beneficial effect of the diet (Reemst et al., 2022a). Furthermore, early supplementation with the essential micronutrients choline, folic acid, methionine, zinc, and vitamins B6 and B12, given from P2 until P9, restored LBN-induced depletion of brain and plasma methionine and rescued adult cognitive impairments as confirmed in hippocampus-dependent tasks (Naninck et al., 2017).

Understanding the mechanisms by which nutritional interventions exert their effects poses a major challenge, because most of the nutrients will have a broad impact on the brain as they are essential building blocks as well as signaling molecules, acting often as co-factors in biochemical processes in the various cell types in our brain, rather than targeting a specific brain region or cell type. In addition, to add another layer of complexity, several of them act on converging pathways, and there is ample cross-talk between the mechanisms modulated by specific nutrients. The effects of nutritional interventions could be, at least partly, mediated by the mitigation of ELA-induced HPA-axis hyperactivity. For instance, early intervention with essential methyl donor micronutrients prevented the ELA-induced increase in CORT at P9 and the associated adrenal hypertrophy (Naninck et al., 2017).

### 3.3 Concluding statement about the importance of the environment

Natural variations in the quantity and quality of maternal care (a major environmental factor in the pups’ early environment) affect later-life neuronal properties and brain function. Importantly, these variations occur even *within* litters, i.e., despite genetic homogeneity and comparable environmental background (apart from maternal care). Careful monitoring of individually received care, therefore, gives valuable information, although such monitoring is cumbersome and labor-intensive. Natural variations in maternal care can be overridden by severe early-life adversity. Particularly, the unpredictability of maternal care in laboratory settings seems a relevant factor that should be monitored at least during the first postnatal week. Nutrition, particularly breastmilk composition, also appears to be a crucial factor in shaping the later-life consequences of ELA, offering promising leads for exogenous intervention. Together, peri-ELA tactile, nutritional, and hormonal stimuli shape the peripheral and central phenotypes accompanying ELA, with lasting consequences for brain function and behavior. Importantly, the early-life environmental factors discussed in this section—and by corollary, interventions thereof—are largely interconnected. It is, therefore, not simple to designate one factor as more important than another, because interfering with one element almost inevitably affects other elements. Yet, in laboratory settings (as discussed above), the various aspects *can* be experimentally varied.

The crucial importance of (monitoring) variations in the early-life environment does not negate the relevance of post-weaning conditions, of which the peri-pubertal phase provides a sensitive window for intervention. More studies are needed to establish if effective interventions involve preventive, normalizing, or compensatory mechanisms.

## 4 The importance of read-out parameters later in life

**Fig. 2:**
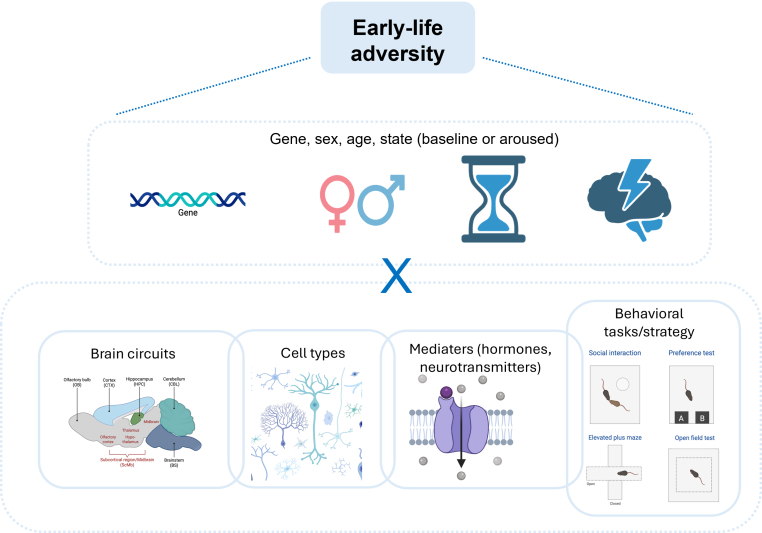
The importance of integrating read-out parameters. Exposure to early-life adversity can alter many aspects of later-life brain function and behavior, although experimental readouts often focus only on a limited set of variables. We advocate for a whole-brain, unbiased approach, preferably one that takes functional circuits (rather than isolated regions) into account. For a more complete insight into the sequelae of ELA, multiple cell types and mediators (transmitters, hormones, immune messengers, etc.) should be investigated. Preferably, more elaborate behavioral tasks than those often used, e.g., allowing the organism to select different strategies to address the situation at hand, are applied. Importantly, the eventual outcome will interact with the individual's sex, age, and genetic background. Also, it is insightful to study organisms not only under baseline but also under aroused conditions, which may probe the functionality of a different set of circuits and mediators. Figure created using Biorender.

Given that ELA exposure in rodents has been intensely studied to understand early adversity as a risk factor for individuals' vulnerability to psychiatric disorders, it is logical that most focus has been so far on genes, molecules, brain areas, circuits, and cognitive domains that are thought to be important for such disorders. However, such focus on a limited set of cell populations, signaling molecules, circuits, and testing conditions (including the array of behavioral tasks used to probe animals’ performance in certain cognitive domains) might have restricted our insights into the lifelong consequences of ELA exposure on also other aspects of brain and its function (Fig. 2) and might have led to an incomplete view of the plethora of changes. We here present a few examples of the impact of ELA on these different levels, encouraging future research to broaden the focus and integrate the existing findings from these multiple levels.

### 4.1 Cell types, brain circuits and mediating molecules, and behavioral domains

*Cell types*: At the cellular level, the focus has been very much on neurons only. However, more recent studies point to an important role of astrocytes, microglia, oligodendrocyte and interactions between these cell types in mediating ELA effects on the brain (Warhaftig et al., 2023). Thus, there is evidence that ELA impacts mouse astrocytes (Abbink et al., 2020; Kotah et al., 2024), even though this is currently based on relatively basic immunohistochemical evidence; whether and how this affects astrocytic *function* remains to be determined. Concerning microglia, there is more substantial evidence of ELA effects in the short-as well as the long-term, regarding morphology, transcriptional profile, and phagocytic capacity (Ahmed et al., 2024; Bolton et al., 2022; Delpech et al., 2016; Hoeijmakers et al., 2017; Reemst et al., 2022b). It has been shown that such ELA-induced priming of microglia also affects how these cells respond to a secondary immune challenge (Reemst et al., 2022b), which might contribute to the increased risk of psychopathologies (e.g., depression and Alzheimer's disease) that notably have a strong neuroimmune component (Herman et al., 2019). Concerning oligodendrocytes, this is still a largely unexplored area, although there is some initial evidence that ELA impacts on myelin (Duan et al., 2025a; Nichols et al., 2025).

This highlights the need to embrace the heterogeneity in the brain and to broaden our focus to the large array of cell types in the various brain regions of interest (see below) and study them in an as much as possible, integrated fashion rather than in isolation.

*Brain circuits*: In terms of brain regions, several of them have been investigated, mainly including those involved in anxiety, memory formation, decision-making, and reward processing, like the hippocampus, amygdala nuclei, prefrontal areas, hypothalamus, VTA, striatum, and nucleus accumbens. It is of course key to realize that these might not be the only ones affected. For instance, a recent genetic tagging study pointed to the paraventricular nucleus of the thalamus as an important player in translating mouse ELA experiences into lasting functional changes (Kooiker et al., 2023). In addition, these areas most likely do not operate in solo but rather as part of specific circuits. Therefore, additional attention to if/how the observed changes in the individual brain region might contribute to changes at levels of circuits and networks will be a key next step to gain further insights into their specific role in mediating the impact of ELA and in identifying optimal target regions for potential interventions.

*Mediating molecules*: Apart from a focus on specific cell types, the field has also concentrated on a limited set of mediators. Thus, ELA undoubtedly activates the pups' stress system (see above), which makes actors of the HPA-axis, specifically corticosterone and CRH, likely mediating molecules. There is a substantial body of literature confirming this in humans (Filetti et al., 2024; Wesarg et al., 2020), including the role of epigenetic modifications of the glucocorticoid receptor (Turecki and Meaney, 2016). Also, molecules important for cognitive processing, including processes thought to be relevant for psychopathology, such as glutamate, GABA, noradrenaline, dopamine, and serotonin have been heavily investigated. But this doesn't rule out other molecules that may mediate ELA effects. For instance, a recent meta-analysis underscored the relevance of sustained increases in pro-inflammatory cytokine signaling after ELA induced by IL-1β, IL-6, and TNF-α across various brain regions (Lumertz et al., 2022).

*Behavioral domains*: Similarly, the angle of ELA models contributing to our insight into psychopathology has somewhat narrowed the battery of behavioral tests applied in rodents. Most studies focused on cognition, anxiety, reward processing and social interaction related behaviors. While these already give us quiet some insights, moving forward will be important to broaden our focus to additional aspects of behavior to other areas like for example eating behaviors, sleep and other underexplored areas in this field. These investigations might have been limited also by the intrinsic limitation of the classically used behavioral paradigms and by the human-biased interpretation of these test. Straightforward and (relatively) standardized as these tests may be—which in principle is a great asset—most of them leave the rodent very few options to choose an appropriate behavioral strategy, which hampers comprehensive insight, particularly as corticosterone is known to change e.g. learning strategies in a sex-dependent manner, both in rodents and humans (Schwabe et al., 2022; Torres-Berrío et al., 2026). Notably, the use of a relatively limited set of behavioral tests does not apply only to the field of ELA studies but also more generally (Torres-Berrío et al., 2026). Interestingly, novel, more in-depth analytical tools hold exciting promise for the future (Jurek et al., 2025; Kovarova et al., 2025). With the development of advanced tracking tools and post-tracking analysis software shared within the research community, new possibilities open up to discern behavioral patterns in experimental and homecage settings (Bordes et al., 2023), enabling the assessment of more complex social behavior (Curley and Champagne, 2023). Instead of only relying on fixed task outcomes, more components of behavior and broader phenotypes and coping styles can be revealed. This is much needed in the translation of human conditions to mouse phenotypes, as human mental health issues are also defined by a multifactor phenotype, as e.g., described in DSM criteria for depression (von Mücke-Heim et al., 2023).

### 4.2 The adult state of the animal in light of an ELA history

The state of the individual when tested in adulthood is of crucial importance to assess the outcome. Thus, the degree of arousal or stress during testing is essential in determining the outcome, as a history of ELA may alter how the brain and the whole organism responds to new challenges. For instance, adult male rats exposed to 24h MD at P3 exhibited *impaired* spatial memory in non-arousing tasks but *improved* synaptic plasticity and emotional learning in aroused/stressed conditions (Oomen et al., 2010), resembling the condition-by-state-interaction earlier observed for the offspring of low (compared to high) licking-grooming rat mothers (Champagne et al., 2008). These differential effects, depending on the state of the animal during testing (including when caused by the task itself), also transpired from an extensive meta-analysis, showing that ELA *impairs* contextual learning under non-aroused conditions but *improves* contextual learning in tasks that involve an acutely aroused state, e.g., fear conditioning (Bonapersona et al., 2019). The distinction between an aroused or non-aroused state is highly relevant, as e.g. exemplified by a later meta-analysis on hippocampus-dependent learning, where such a distinction was not specifically made (Rocha et al., 2021).

**Fig. 3:**
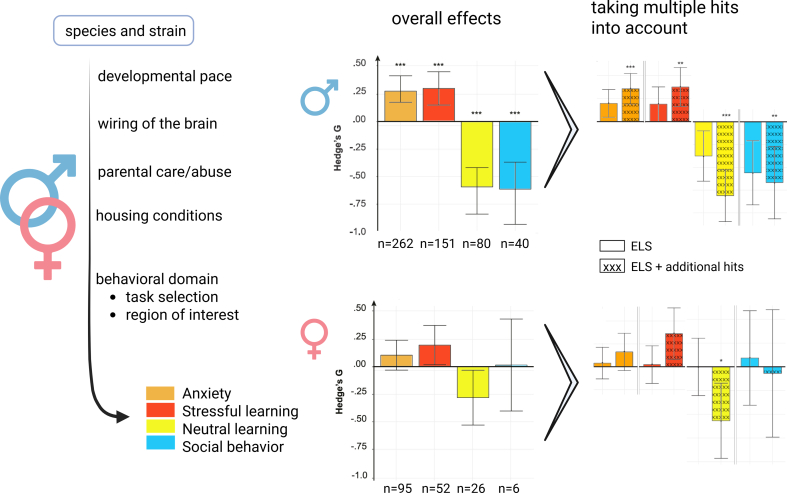
The importance of sex differences. Differences in the sequelae of ELA between male and female rodents depend on multiple variables that can play a role during various periods across the lifespan. During (early) development, the wiring of the brain differs between males and females, as well as the pace at which particular brain regions and circuits mature. Also, characteristics of parental (e.g., maternal versus biparental) care can differ between male and female pups, with, in general, more care bestowed on the male than female offspring and a higher likelihood of abuse toward the female (compared to the male) offspring. Post-weaning housing conditions like group vs single housing can have different effects on males and females. When testing the consequences of ELA, the set of brain areas and (behavioral) tests that are chosen may also contribute to the differences observed between male and female offspring, especially because many tests are optimized for male organisms, e.g., those examining spatial memory formation. Sex differences can also vary according to species and strains used. (Bar graphs) In general, female offspring exposed to ELA are heavily understudied compared to the males (see main text for details). Nonetheless, a meta-analysis demonstrated that male and female offspring differ in the effect size (Hedge's g-score) rather than the directionality of behavioral changes observed after ELA exposure, at least in the four behavioral domains included in this meta-analysis. ELA effects on behavior are amplified by multiple hits in both sexes. (n) depicts the number of studies included per domain. Adapted with permission from Bonapersona et al. (2019).

The condition-by-state interaction not only pertains to the acute situation during testing but also seems to occur when rodents are exposed to a chronic stressor in adulthood against a background of ELA. Thus, Peña et al. (2019a) showed that mice exposed to ELA between P10 and P17 had longer latencies to eat in an open arena after a 3-day period of subthreshold stress in adulthood, but comparable behavior to controls without this additional stress period. More generally, they found that early-life stress sensitizes the response to future stressors at the behavioral, neural circuit, and molecular level (Peña, 2025). Similarly, microglia from ELA-exposed mice respond differently to a later-life immune challenge (LPS) in terms of gene expression profiles compared with microglia isolated from control mice (Reemst et al., 2022b). Overall, these findings are consistent with the observation that ELA effects on behavior are amplified by multiple hits (Bonapersona et al., 2019, see also Fig. 3), especially in combination with a vulnerable genetic background (Daskalakis et al., 2013). Yet, other studies have described that adverse conditions early in life may “inoculate” animals against later-life stress (Ashokan et al., 2016; van Doeselaar et al., 2025) or at least prepare them to function well when early and later life conditions match (Champagne et al., 2009; Schmidt, 2011). Therefore the animals’ state and the context are essential for the interpretation of the impact of ELA.

Another aspect involving the animal's state is the time of day at which it is tested, as many hormones, including corticosterone, circulate in circadian and ultradian rhythms (Lightman et al., 2020). Different results might be obtained in the active compared to the inactive phase of mice (see e.g. den Boon et al., 2019; Parfitt et al., 2007). This is reminiscent of early work in humans showing that corticosteroids increase the *threshold* for sensory information during the inactive phase, while they improve the *interpretation* of sensory information during the active phase (Henkin and Daly, 1968). This is now highly unstandardized as impact of ELA is not consistently tested during the same time phase of the day, hampering comparability across studies.

### 4.3 Concluding statement about the importance of read-out parameters later in life

To better understand the lasting consequences of ELA exposure, it is beneficial to be as comprehensive and integrative as possible with regard to *i)* the array of cell types, ii) the brain regions and networks to be studied after ELA and the *iii)* the mediating molecules. Moreover, we argue that, next to the standard behavioral tests currently used, *iv)* more elaborate tests should be employed, allowing the animal to choose from a repertoire of behavioral strategies, the use of unbiased behavioral observations as well as introducing tests that probe behavioral domains outside those directly associated with psychopathology. Finally, to get a better picture of the consequences of ELA, the experimental design should allow examination of the animal's adaptive capacity under baseline *and* more challenging conditions, preferably during the active and inactive phases of the day. These considerations (summarized in Fig. 2) come in addition to the notion that read-outs can be influenced by sex (see below), age, and genetic background (not discussed).

## 5 The importance of studying both sexes

Many of the current ideas on how ELA affects rodent brain development are based on males, while there are important sex differences at play during development (Fig. 3 left side). Meta-analyses focusing on brain effects of postnatal stress report that ∼75% of all animals included are male (Bonapersona et al., 2019; Reemst et al., 2022c; Schuler et al., 2022); the percentage of *studies* involving exclusively males is even higher (Duan et al., 2025b; Lumertz et al., 2022). The reason often put forward is that studies in males circumvent the variability introduced by the estrous cycle. However, while the impact of sex hormones on the brain is undeniable (Rocks et al., 2022), there is no convincing evidence for enhanced variability in females (Becker et al., 2016). Below, we first discuss whether differences in ELA outcomes between males and females are merely quantitative or also qualitative. And next, address how the experimental design regarding early-life environment and later-life readout tests may contribute to the observed differences.

### 5.1 Qualitative and/or quantitative differences?

Superficial examination could lead to the conclusion that the differences between the adult brains of males and females exposed to ELA involve a difference in effect size (quantitative) rather than directionality (qualitative). More specifically, at a meta-analytical level, the directionality of ELA effects on adult behavior was found to be similar in male and female (adult) offspring, yet the effect sizes in females were smaller than in males (see Fig. 3, bar graphs overall effects). In males, anxiety-like behavior was clearly increased and performance on stressful learning tasks improved, while performance on non-stressful learning tasks was impaired and social behavior diminished, while in female effects were less strong. In both males and females effect sizes were evidently larger when additional stressors or ‘multiple hits’ (e.g. prenatal stress due to transport of pregnant females, chronic individual housing or stress prone strains) were present in a study (Fig. 3, bar graphs when multiple hits are taken into account) (Bonapersona et al., 2019). The smaller effect sizes in females might be partly attributable to the lower number of studies, in combination with low sample sizes, a common problem in experimental animal studies, causing issues about the reproducibility of findings (see e.g. (Bonapersona et al., 2021; Button et al., 2013; Carneiro et al., 2018; Jordan et al., 2026)).

However, male-female differences do not only exist with respect to effect sizes. In fact, some have argued that sex differences in the directionality of the effects may be the rule rather than the exception (Bath, 2020). In agreement, several studies have reported effects observed only in males or only in females (Bath, 2020; Cattaneo et al., 2024; Kundakovic et al., 2013; van Doeselaar et al., 2025), or even opposite effects in male versus female offspring after ELA exposure. For instance, at the cellular level, 24h MD at P3 increased rat hippocampal neurogenesis in male offspring at P21, but decreased it in females (Oomen et al., 2009). In another study, LBN exposure in mice reduced neurogenesis at baseline in male but not females at 4 months of age (Naninck et al., 2015). Yet, exposure to prolonged exercise revealed a lack of enhanced neurogenesis in LBN-exposed female mice, suggesting that exposure to a second hit unmasked a latent effect in females that was not apparent at baseline (Abbink et al., 2017). At the behavioral level, clear disparities between male and female rats were observed in the LBN model regarding motivation to work for a reward, depending on the nature thereof (Williams et al., 2022).

### 5.2 Sex-dependent differences and maternal care

How can the early-life environment contribute to male-female differences? On average, male pups receive more care from the dam than females, with a higher degree of anogenital licking, which is hypothesized to contribute to psychosexual differentiation (Moore, 1992). The sex-dependent difference in received maternal care may, to some degree, also explain why the lack of stimulation due to ELA exposure has a greater impact on male development: This argument has often been used by authors to explain the more potent effects of ELA observed in males. It might be rooted even deeper, though: From an evolutionary point of view, sex differences in the impact of ELA might stem from a conflict between parental and offspring genes, with male offspring having a greater genetic conflict with their mothers than female offspring, thereby contributing to sex specific vulnerability (Furness and Pollux, 2025). Yet, others have shown that females tend to receive more maltreatment from stressed dams, and this could explain their results of female rats being more influenced by ELA compared to males (Keller et al., 2019).

Most likely, the results depend on the type of models and strains as well as on the specific outcome of interest (see below). For instance, in mouse strains with biparental care, disruption of paternal care was found to particularly induce impairments in the female offspring, whereas models focusing on maternal care appear to affect male offspring more than females (Walker and Glasper, 2025). Also studies including different models in the same lab, find differential outcomes, e.g. in C57BL/6N mice, the LBN model affects physical growth and timing of developmental milestones in both sexes, while only females showed later life anxiety-like behavior, while the MS with isolated pups model also affected both sexes similarly in developmental milestones, yet only males showed changes in stress physiology and anxiety-like behavior (Demaestri et al., 2020). All in all, the jury is still out, but it seems very likely that the specific experimental protocol can have important effects on male-female differences, underscoring the importance of documenting litter composition and maternal care, as well as directly comparing the two sexes within the same study.

### 5.3 Sex-dependent differences in developmental trajectories

Some of these sex-dependent differences could be partly explained by the fact that male and female brains are differently wired and have different developmental trajectories (Di Trapano et al., 2025; Le et al., 2022; McCarthy et al., 2012). The latter is highly relevant in relation to the selection of ELA models, e.g., during the first versus the second postnatal week. Thus, female rodents, compared to males, show a faster developmental trajectory (Brouwer et al., 2021; Premachandran et al., 2020), so that exposure to stress during the first postnatal week “hits” the brain at a different stage and might have less impact e.g. on hippocampal and amygdala function, whereas one could expect more apparent effects in cognitive domains related to PFC function (Lupien et al., 2009). In agreement, adult females, but not males, with a history of LBN rearing not only show more depressive-like behavior, deficits in attentional learning, accelerated amygdala maturation, delayed sexual maturation, but also impaired contextual fear expression, and delayed development of particular classes of cells in the PFC (Bath, 2020). Conversely, males with an LBN history rather have impaired development of object location learning, accelerated hippocampal and amygdala development, and shifts in the timing of cued and contextual threat learning. To truly compare the impact of ELA on the brains of females and males, one would need to target comparable developmental windows, which is complicated, as not all regions develop at the same pace.

The impact of ELA in males versus females also depends on the task selected to probe the later-life consequences. Indeed, it is well-documented that females and males are differently affected by ELA exposure depending on the (cognitive) domain tested (Kundakovic et al., 2013; Millstein and Holmes, 2007; Reemst et al., 2022c). This may partly result from the sex-dependent strategies employed in behavioral tasks, as well as the fact that many adult behavioral readouts are optimized for males. The latter is nicely illustrated by studies using the Barnes maze, in which male mice use a predominant spatial strategy, shifting toward more stimulus-response learning under stressful conditions (Schwabe et al., 2008), while females are more stimulus-response-oriented during basal conditions and favor spatial learning under stress (ter Horst et al., 2013). Depending on the availability of spatial and stimulus-response cues, one might miss or misinterpret the results.

### 5.4 Concluding statement about the importance of studying both sexes

Until now, female rodents exposed to ELA have been heavily understudied, even though it is generally acknowledged that addressing sex as a biological variable in preclinical research is important and feasible (Dalla et al., 2024). Since development in general, and brain development in particular, as well as environmental factors, such as the parent-pup interactions, are sex-dependent, we can expect that comparable ELA paradigms will result in sex-dependent changes in the brain and neuroendocrine system. Preferably, male and female offspring should be studied in the same experimental series, monitoring differences from the earliest moment (e.g., sex-dependent difference in maternal care) to the moment of testing the consequences in adulthood. In general, the field would benefit from varying i) the ELA paradigms, and ii) the readouts so that they are optimized to probe potential effects in the brain of females, which will provide a valuable and indispensable extension of the current ideas on how ELA lastingly alters brain function.

## 6 Discussion

This contribution highlights several aspects one might want to consider when designing a rodent study investigating the long-lasting effects of ELA exposure (see box 2). Rather than zooming in on specific aspects, we have chosen a helicopter view, touching on a great variety of experimental aspects. For more detailed considerations on specific aspects of ELA, we refer to excellent reviews by others, e.g., Bath (2020), providing a synthesizing view on sex differences in response to ELA; or Jorgensen et al. (2025), providing a systematic review of animal models in relation to later-life consequences of ELA.

Box 2 **Some recommendations for the experimental design of ELA studies**

Below, we summarize a few recommendations for the design of reproducible ELA experiments. The listed suggestions are global and come on top of the specific elements described in sections 2, 3, 4, 5. This list is by no means exhaustive and will require a collective effort by the field i) to reach consensus on the key aspects impacting outcome, ii) to always carefully report all relevant experimental details and, if possible iii) to ultimately develop a roadmap for experimental ELA studies.

When designing ELA experiments and placing your findings in the context of the existing literature it is important to take into account several aspects:

• Related to timing: It is key to consider the developmental stage during which the exposure to early-life adversity takes place, the duration and frequency of the early life adversity exposure, the age and time of the day during which the outcome parameter is studied and the time lapse between exposure and outcome assessment.
• Related to environmental aspects: It is key to consider nutrition, temperature, and nest and housing conditions during early-life exposure as well as during the assessment of the outcome parameter of choice.
• Concerning behavior: As to maternal care, it is important to realize the effects of the ELA exposure on (predictability of) maternal behavior. Monitoring mother-pup interactions is important, when possible even at the level of individual pups, as intra-litter variation in maternal care has been shown to impact later life outcomes. Concerning behavioral assessment in the offspring, it is advised to integrate, when possible, multiple behavioral domains as well as the implementation of novel tracking tools to gain further insights into the impact of ELA on behavioral patterns over time.
• Concerning sex: Preferably, experimental series should be carried out both in males and females, and include as many readout parameters and time points across the developmental trajectory as possible and feasible.
• Concerning sample size: A well-powered study either through the use of sufficiently large sample size and/or with the use of historical data (e.g. for the controls when possible) is essential for the generation of reproducible data.

Alt-text: Box 2

In short, we argue that study designs preferably pay attention to the early-life environment (particularly the nature of the parent-infant interactions); multiple time points during development; post-weaning conditions; various networks and cognitive domains, including but not restricted to “the usual suspects”; and both sexes. Sufficient statistical power needs attention (Bonapersona et al., 2021; Button et al., 2013; Carneiro et al., 2018; Jordan et al., 2026), although meta-analytical approaches partly address this issue.

A well-powered study generally asks for considerable sample sizes, well beyond the usual 6-10 animals per experimental group. In the current literature this is most often not met due to practical limitations, impacting reproducibility. It has been proposed that power can be enhanced by reusing historical data, especially for control groups and common experimental endpoints (Bonapersona et al., 2021). This needs to be done with caution and it is only possible when all the other aspects of study design (see above) allow for such approach. Moreover, we cannot stress the quality of reporting enough; a detailed reporting of the methodology and lab environment can help to explain differences observed between laboratories, increase reproducibility and fine-tune ELA models, see also the ARRIVE guidelines for good and transparent reporting (https://arriveguidelines.org).

Although studying the effects of a challenging environment on the developing rodent brain is of interest in its own right, these paradigms are often selected to better understand the etiology of ELA as a documented risk factor for the later-life onset of mental disorders in humans, given their clear advantages (next to the obvious disadvantages), i.e. i) excellent control over genetic and environmental factors, ii) the short duration from conception to (young) adulthood which allows investigation of causality and multiple generations, and iii) the possibility to examine the underlying neurobiological mechanisms in great detail. One may wonder if these three advantages have been fully exploited.

Regarding the first point, i.e., excellent control over/insight into the early-life environment, detailed observations of mother-pup interactions have seldom been taken into account. A good example of the value of such detailed observations is the description of high-level entropy in dams living in LBN conditions (Molet et al., 2016), which has guided subsequent experiments in human cohorts (Birnie and Baram, 2025; Davis et al., 2017). Such detailed observations are cumbersome, yet very informative. Moreover, the fact that most studies have been restricted to models of maternal (and not paternal) care (Walker and Glasper, 2025) and have not compared the consequences of ELA across multiple developmental windows has limited our insights.

With respect to understanding causal relationships, the possibilities of ELA models have remained underexploited. Ideally, study designs should allow for longitudinal investigations so that differential effects can be observed across the entire lifespan. This is challenging, especially in the context of aging research, but it is also very informative (Kotah et al., 2021; Workel et al., 2001). Causal relationships have often been inferred from the effectiveness of genetic, pharmacological, or nutritional interventions in preventing or normalizing ELA-induced changes (Cattaneo et al., 2024). This may be deceptive, though, because such interventions may change brain function in their own right, counteracting rather than directly interfering with cascades caused by ELA exposure (Holloway and Lerner, 2024).

Admittedly, many studies in animal models have taken advantage of the ability to examine, in great neurobiological detail, the consequences of ELA exposure on brain function. This has resulted in an avalanche of detailed knowledge at the genetic, epigenetic, cellular, circuit, and behavioral level, in neurons and -more recently- (micro)glial cells, involving neurotransmitters and peptides, but also mediators that link the brain to the rest of the body, such as adrenal steroids or inflammatory agents. While this has resulted in a steep increase in publications and yielded many valuable insights, most studies have been restricted to brain regions and systems thought to be relevant to human psychopathology. Clearly, this has resulted in a rather incomplete picture.

The possible relevance of ELA sequelae for human psychopathology has been a major driver for many of the rodent model studies discussed above, although this was not the case for the founding studies by Gig Levine in the 1950s (Levine, 1957). The latter is of relevance, also because the consequences of ELA are not universally negative. They may serve to predict and prepare organisms for comparable challenging conditions later in life (McLaughlin et al., 2021; Nettle et al., 2013; Rickard and Lummaa, 2007), with potentially beneficial effects for the individual (and species) when early-life and later life conditions match, as also observed in studies using rodent models (Branchi and Cirulli, 2014; Champagne et al., 2009; Schmidt, 2011). Whether or not the consequences of ELA in humans will increase the likelihood to develop a mental disorder depends on many other factors, including severity and chronicity of ELA, genetic susceptibility, later life environment, and the dynamic ability to learn how to adapt, which is thought to be crucial for resilience, i.e., the potential to stay healthy in the face of adverse conditions (Hermans et al., 2025; Rutter, 2012). Animal models could be even more valuable if aspects of vulnerability and resilience are both taken into account in the study design, although interpretation of those aspects should be done with caution: Whether a change in behavior represents a sign of vulnerability or resilience is not always easy to interpret in a laboratory setting, where survival is mostly not at stake. In principle, the organism's response to (early-life) stress is, within limits, adaptive in a context- and state-dependent manner (Krugers and Joëls, 2025).

In conclusion, carefully designing ELA studies is 90% of the work, the adage of Seymour Levine. Standardized protocols have clear advantages, e.g., allowing comparisons across labs. However, varying protocols and readout parameters and then comparing results between—or within—labs can provide an even richer view of the lasting consequences of ELA exposure on the brain.

## CRediT authorship contribution statement

**Rixt van der Veen:** Conceptualization, Visualization, Writing – original draft, Writing – review & editing. **Marian Joëls:** Conceptualization, Visualization, Writing – original draft, Writing – review & editing. **Aniko Korosi:** Conceptualization, Visualization, Writing – original draft, Writing – review & editing.

## Declaration of competing interest

The authors declare that they have no known competing financial interests or personal relationships that could have appeared to influence the work reported in this paper.

## Data Availability

No data was used for the research described in the article.
